# Mapping the resistance landscape: A large-scale study of polymyxin-resistant pathogens circulating in low-and middle-income countries

**DOI:** 10.21203/rs.3.rs-10465197/v1

**Published:** 2026-08-03

**Authors:** Tania Da Silva Duarte, Holly E. E. Floyd, Visanu Thamlikitkul, Van Dinh Trang, Pham Ngoc Thach, Cely Abboud, Ana Cristina Gales, Fernanda Fernandes dos Santos, David Ojok, Gopi Aryal, Mahesh Kumar Chaudhary, Abdul-Wahab Omo-ope Ettu, Abdulakeem Adams, Mohamed Adel Hassan, Ahmed Mohamed Omar, Yasra Sarwar, Ian Morrissey, Richard Alm, Tanuka Sen, Alysha G. Elliott, Matthew Cooper, Mark B. Blaskovich, Johannes Zuegg

**Affiliations:** The University of Queensland; The University of Queensland; Mahidol University; Hospital for Tropical Diseases; Hospital for Tropical Diseases; Instituto Dante Pazzanese de Cardiologia; Universidade Federal de São Paulo; Universidade Federal de São Paulo; Centre for Infectious Disease Research in Zambia; Nepal Mediciti Hospital; Nepal Mediciti Hospital; Innovation Curae Limited; Innovation Curae Limited; City of Scientific Research and Technological Applications; City of Scientific Research and Technological Applications; National Institute for Biotechnology and Genetic Engineering; International Health Management Associates; Combating Antibiotic-Resistant Bacteria Biopharmaceutical Accelerator; The University of Queensland; The University of Queensland; The University of Queensland; The University of Queensland; The University of Queensland

## Abstract

The increasing prevalence of antimicrobial resistance is a major public health challenge, particularly in low- and middle-income countries (LMICs). Polymyxins are last-resort antibiotics used for treating highly drug-resistant infections, however, the rise of polymyxin-resistant bacterial strains is further reducing treatment options in LMICs, where the burden is exacerbated by limited diagnostic capacity, poor antimicrobial stewardship, and limited surveillance infrastructure. There is a lack of comprehensive population-based surveillance of the emerging polymyxin resistance and a need to comprehend what genetic determinants are associated with this resistance. In this study, we collected 634 clinical isolates of polymyxin-resistant bacteria from 28 LMICs, then used whole genome sequencing, phylogenetic and bioinformatic analyses to identify species, sequence types and antibiotic resistance gene profiles. We found 12 bacterial species and focussed downstream analyses on 4 high priority pathogens: *K. pneumoniae, E. coli, A. baumannii, and P. aeruginosa*.. The analysis revealed clonal expansion of high-risk lineages across geographically dispersed LMIC settings. Phenotypic antimicrobial susceptibility testing using both VITEK 2 automated systems and broth microdilution (BMD) assays against an expanded panel of 44 antibiotics allowed us to correlate the bioinformatic analyses to the resistance profiles. These findings show clonal spread and horizontal gene transfer feature in the propagation of antimicrobial resistance and highlight that enhanced genomic surveillance is essential to inform treatment strategies and mitigate the spread of resistance to last-line antimicrobials.

## INTRODUCTION

Antimicrobial resistance (AMR) is one of the most critical global health threats of the 21st century, threatening effective treatment of bacterial infections and undermining decades of medical progress^1,2^. The World Health Organization (WHO) has highlighted AMR as a priority public health crisis, with projections suggesting that drug-resistant infections could cause 10 million deaths annually by 2050 if left unchecked^3^. The burden is particularly severe in low- and middle-income countries (LMICs), where constrained healthcare infrastructure, limited diagnostic capacity, and weaker antimicrobial stewardship frameworks exacerbate the problem^4,5^.

Polymyxin lipopeptide antibiotics, including colistin (polymyxin E) and polymyxin B, are among the few remaining therapeutic options for infections caused by multidrug-resistant (MDR) and extensively drug-resistant (XDR) Gram-negative pathogens^6,7^. First discovered in the 1940s then largely unused due to nephrotoxicity concerns, a clinical resurgence has been driven by the global dissemination of carbapenem-resistant Enterobacterales, *Acinetobacter baumannii*, and *Pseudomonas aeruginosa*^8^. However, the emergence of polymyxin resistance in these organisms now threatens to undermine its utility as a last-resort antibiotic^9,10^, especially in those LMIC’s where newer drugs, like cefiderocol are not readably available or unaffordable, and the ‘old’ drug colistin is applied more frequently. Resistance mechanisms include chromosomal mutations in two-component regulatory systems (e.g., *pmrAB*, *phoPQ*) and the acquisition of mobilised colistin resistance (*mcr*) genes via plasmids, which facilitate horizontal gene transfer across bacterial populations^11–13^.

Despite the clinical importance of polymyxin resistance, surveillance remains fragmented and uneven, particularly in LMICs. Existing studies are often geographically restricted, species-specific, or limited in sample size, leaving critical gaps in understanding the population-level epidemiology and genetic basis of resistance^14–16^. LMICs often do not collect and store clinical isolates, and polymyxin resistance is often not included in standard antimicrobial testing. Standardised AST systems, such as VITEK 2, often exclude polymyxin from AST cards, and studies have shown that automated systems can have unacceptable error rates^17–19^. This scarcity of comprehensive data restricts the ability to track clonal dissemination of high-risk lineages, detect novel resistance determinants, and inform targeted interventions.

Genomic epidemiology offers unprecedented resolution in AMR surveillance, allowing simultaneous identification of bacterial species, resistance determinants, and clonal relationships^20,21^. Whole genome sequencing (WGS) has proven instrumental in characterising resistance mechanisms and mapping transmission dynamics across diverse settings^22^. However, large-scale WGS-based analyses of polymyxin-resistant pathogens remain rare in LMIC contexts, despite these regions being recognised as hotspots for resistance emergence and spread^23^.

To address these knowledge gaps, we conducted a large-scale genomic study of 634 polymyxin-resistant clinical isolates collected from 28 LMICs during 2021–2022, with some samples collected up to five years earlier but sent to us during this period. Using WGS, phylogenetic analyses, and phenotypic antimicrobial susceptibility testing (both Vitek 2 and BMD against 44 antibiotics), we investigated species distribution, sequence types, and resistance gene profiles, with a particular focus on four WHO-designated priority pathogens: *Klebsiella pneumoniae*, *Escherichia coli*, *A. baumannii*, and *P. aeruginosa*. Our findings highlight clonal expansion and horizontal gene transfer as drivers of resistance in LMICs and underscore the urgent need for enhanced genomic surveillance to preserve the efficacy of last-line antibiotics.

## MATERIAL & METHODS

### Sample collection

The isolates were collected by each site, using their own protocols and ethics for collection, isolation and characterisation. The aim of the collection was to select for Gram-negative pathogens that i) represent species constituting the current clinical challenges for that country, and ii) display a high level of resistance, multi- or pan-resistance against commonly used antibiotics, with special interest in any isolates resistant against polymyxin B or colistin. Each collection site performed the isolation, identification, characterisation and shipment according to their best practice (see Table 1), shipping selected isolates to the University of Queensland site in Brisbane (Australia).

The in-country collections were supplemented with the addition of 92 isolates from the IHMA (International Health Management Associates, Monthey, Switzerland) collection. The selection included Gram-negative bacteria, from LMIC with a multi-drug resistance profile, preferably with resistance against colistin or polymyxin. The IHMA isolates were shipped to University of Queensland as glycerol stocks, as dry-ice shipment.

#### Sample Preparation and Storage

All samples were prepared into sets of glycerol stocks, one for frequent use and one for long-term storage of the isolates (Supplementary Figure S1). Upon arrival (Day 1), the isolates were struck out on Tryptic Soy Agar (TSA) (BD, Bacto Laboratories) and incubated overnight at 37°C. The following day (Day), each plate was visually inspected for purity and a single colony of pure culture was struck out again on TSA and incubated overnight at 37°C. For the plates with different colony morphology, single colony from each morphology was struck out on different TSA plate. On Day 3, a pure colony from each plate was inoculated in 4 mL of Trypticase Soy Broth (TSB) (BD, Bacto Laboratories) and incubated at 37°C overnight, on an orbital shaker at medium speed. For samples which were reported as polymyxin B or colistin resistant by the collection site, polymyxin B (Sigma Aldrich) was added to additional agar plates and the broth solution, at a final concentration of 2 μg/mL. For those samples, the antibiotic-free plates and broth were used further in case the 2 μg/mL polymyxin plates or broth did not display sufficient growth. Glycerol (Sigma Aldrich) was added to the overnight cultures for a final concentration of 20% glycerol, and aliquoted into the respective vials, vortexed and snap frozen in liquid nitrogen and stored at −80°C. *Neisseria gonorrhoeae* from the IHMA collection were received as glycerol stocks and were transferred to new tubes without regrowth.

#### Antibiogram by Broth Microdilution (BMD) Assays

The MIC of each isolate was tested using the BMD as per the CLSI guidelines^1,2^ (Supplementary Figure S2). The isolates were struck out from the in-house high use glycerol stocks and cultured for 18–24 hours on TSA. The following day, each culture plate was visually checked for purity. The isolates were then inoculated in 4 mL of TSB and incubated at 37°C overnight, on an orbital shaker, at medium speed. For samples which were reported as polymyxin B or colistin resistant by the collection site, polymyxin B (Sigma Aldrich) was added to the culture TSA plates at a concentration of 2 μg/mL in the agar.

Assay-ready test-plates were prepared by Compounds Australia (Brisbane, Australia) by dispensing 44 antibiotics, including 4 antibiotic combinations, into 384-well polystyrene microtitre plates (Corning #3680) as 2 μL droplets of 16 × 1:2 dilutions, using liquid handling robots (see Supplementary Table S1). The solvent used for each antibiotic was based on EUCAST/CLSI guidelines, with some adjustments required based on solubility testing. The test-plates were stored at −80°C and moved to room temperature on the day of the assay. Once thawed, a sample of each overnight culture was diluted 40-fold in Cation Adjusted Muller Hinton Broth (CAMHB) (BD, Bacto Laboratories) and incubated at 37°C for a further 2–3 hours, on an orbital shaker. The resultant mid-log phase cultures were diluted in CAMHB and added to the 384-well test-plates to give a final cell density of 5 × 10^5^ CFU/mL, in a final volume of 50 μL. The test-plates were covered, briefly centrifuged at 800 RPM and incubated at 37°C for 18–24 hours.

For MIC evaluation, the optical density was read for each well at 600 nm (OD_600_) using a Tecan M1000 Pro Spectrophotometer. The MIC value was determined as the lowest concentration at which OD_600_ demonstrated ≥ 80% growth inhibition compared to growth control. Analysis was performed using an in-house analysis and database system, calculating the % growth inhibition for each well, in comparison to the median of the positive (inoculated media) and negative (media only) controls. For isolates which displayed low growth (low OD_600_ readouts) or patchy growth (high standard deviation of positive growth control) in the 386-well test-plates, resazurin (Sigma Aldrich) was added to test-plates at a final concentration of 0.01%, incubated for a further 3–4 h at 37 °C, and analysed by measuring the optical density at 570 and 600 nm and using the difference between OD_570_ and OD_600_ for the calculation of percentage growth inhibition.

For quality control, each test-plate with Z’-Factor < 0.25 was rejected. In addition, each assay run included *E. coli* ATCC 29552 as control strain as per EUCAST/CSLI guidelines, rejecting any MIC values of those antibiotics that displayed an abnormal MIC value (> 2 dilutions from the expected MIC value). These rejections mainly affected antibiotics with low chemical stability in solution (due to degradation during the test-plate preparation) or were caused by liquid dispensing issues.

#### Identification and antimicrobial susceptibility testing (AST) by Vitek2 (bioMérieux)

BioMérieux’s VITEK2 Compact system was used to identify (ID) and perform antibiotic susceptibility testing (AST) of the isolates. Isolates were struck out in TSA with or without polymyxin B, as described in the Sample Preparation section, except for *N. gonorrhoeae* which was struck out in GC agar (Thermo Fisher Scientific). The plates were incubated and Vitek2 cards loaded following the standard Vitek2 protocol. For identification, we used Vitek2-GN (for Gram-negative bacteria) and Vitek2-NH (for Neisseria species). For AST we used AST-N411, AST-N435 and AST-GN96 cards (bioMérieux). All results were saved using the most up to date (2021–2024) EUCAST and CLSI databases and transferred to the in-house database system for analysis.

#### DNA extraction and sequencing

Genomic DNA (gDNA) was extracted initially in-house for the first 270 isolates, with the remaining isolates extracted by the sequencing provider, Australian Genome Research Facility (AGRF, Australia). All DNA samples were sequenced by AGRF using Illumina in one of the following lanes: NovaSeq SP Lane, 300 cycles, NovaSeq S1 Lane, 300 cycle, and NovaSeq X-plus lane. Exception to this sequencing pipeline were the isolates from NHTD (Vietnam) which were processed and sequenced by the collaborator site NHTD (Vietnam).

For in-house gDNA preparation we used inoculums from the glycerol stock, grown in 6 mL of TSB overnight at 37°C, in an orbital shaker at medium speed. The culture was centrifuged at 3,000xg for 5min in a bench centrifuge and the supernatant was removed and discarded. 5 mL of 1x PBS (Thermo Fisher Scientific) was added to the pellet and mixed by inversion, followed by centrifugation as described above. The supernatant was removed and discarded, and the washed pellet dissolved in 200 uL of 1xSTE (Sigma Aldrich) or 1x PBS, and transferred to a 1.5 mL low-binding Eppendorf tube. 200 uL of DNAzol (Life Technologies Australia Pt) was added to the tube, mixed by inversion and incubated in a rotator (Intelli mixed) for 5 min, (mode C2, 14 RPM), following RNase treatment with 20 uL of RNase cocktail (Thermo Fisher Scientific) and incubation of the sample for 15 min at 37°C, followed by addition of 200 uL of chloroform (Merck) and incubation in the rotator as described above. The sample was then centrifuged at 12,000xg for 10 min at RT, and supernatant transferred to a 2 mL Eppendorf tube, adding 3M sodium acetate (pH 5.2) (1:10 sample volume) and 1 mL (approximately 2 volumes) of cold 100% ethanol for precipitation of the DNA, followed by incubation for 3 minutes at RT or at −20°C overnight. To purify and concentrate the DNA, the DNeasy Blood & Tissue Kit (Qiagen) or DNeasy Blood & Tissue 96 plates kit (Qiagen) were used, and the protocol was followed according to the manufacturer’s instructions starting from the step of transfer of the sample mixture to the DNeasy Mini spin columns. 50uL of Buffer AE were used to elute the gDNA. The gDNA was quantified with Qubit^®^ 2.0 fluorometer using the Qubit^™^ dsDNA HS assay kit (Thermo Fisher Scientific), the purity was accessed with a Nanodrop 1000 Spectrophotometer and Nanodrop One (Thermofisher scientific).

### Ethics

The Medicine LNR ethics committee from the University of Queensland human research ethics approved the current study under the project number “2021/HE001290 Collection of multi-drug-resistant bacteria from clinical isolates”. The participants enrolled in the study provided verbal or written informed consent to the country of the sample’s collection.

#### Whole genome sequencing data processing

All bioinformatic analysis were performed at the University of Queensland, utilizing their high-performance computing facilities.

##### Trimming reads:

Paired-end reads from Illumina sequencing were base-called and demultiplexed by AGRF and trimmed and removed from adapter sequencing using *Trimmomatics* (v0.39), using the following parameters ‘NexteraPE-PE.fa:2:30:10:2:TRUE SLIDINGWINDOW:4:15 HEADCROP:20 LEADING:10 TRAILING:10 MINLEN:36’. The quality check of the reads was done by *FastQC* (v0.11.9).

##### Assembly:

The assemblies were built using *shovill* (v1.1.0), using default parameters, and resulting contigs filtered by *filter_contigs.py* from https://github.com/chrisgulvik/genomics_scripts, applying length, coverage, complexity and GC content filters. Any assembly was checked by *CheckM*^24^ (v1.1.3), for completeness, genome size, and contamination.

##### Taxonomy, Sequence- and Sero-Type:

The taxonomic classification was performed using *Kranken2*^25^ (v2.1.3) using the k2_pluspf_16gb database (2022) and *GTDB-Tk*^26,27^ (v2.2.6). The multi-locus sequence typing was assessed using *MLST*^28^ (v2.23.0), while serotype classifications were performed by species-specific tools: *PAst*^29^ (v3) for *P.aeruginosa*, *ECTyper*^30^ (v2.0.0) for *E.coli* and *Kaptive*^31^ (v3.0.0b6) for *K. pneumoniae* and *A. baumannii* sequences.

###### AMR Gene identification

AMR gene identification was performed by *AMRFinderPlus*^32^ (v4.0.19) with database version 2024-12-18.1, and *RGI*^33^ (v6.0.5) using database version 4.0.1, both using default parameters including nomination of specie names. In addition, *Kleborate*^31^ (v3.1.3) was used for *K.pneumoniae*

###### Plasmid, Annotation and Phylogeny

Classification of plasmids were done by *MOB*_*Recon*^34^ (v3.1.9), applying the plasmid identification of contigs to the genome assembly file. Annotation was performed with *Prokka*^35^ (v1.14.6), and calculating phylogenetic trees from genome assembly were performed by *parSNP* (v2.1.1) using an ATCC reference genome for each specie and using *Muscle* for multiple sequence alignment and *FastTree* for phylogeny tree generation.

##### Nucleotide Accession Numbers:

The whole-genome project was deposited at the NCBI GenBank (https://www.ncbi.nlm.nih.gov/genbank/) under BioProject number **PRJNA1322038**.

## RESULTS

### Scope of isolate collection

The aim of collecting a range of isolates from low/medium income countries (LMICs) was to get a current snapshot of multi-drug resistant (MDR), Gram-negative clinical isolates from a geographically disperse set of countries where antimicrobial stewardship is often not as efficiently enforced as in high income countries (HICs). Multi-drug resistance profiles are not only more common but also commonly include resistance against a broader range of antibiotics. In this sense, the primary aim of the collection was create a collection of contemporary highly multi-drug or extreme-drug (XDR) resistant isolates so they could be used in drug discovery and development programs as benchmark isolates to test novel antibiotics against ‘real-life’ isolates from regions where new resistance mechanism often arise. The countries were not systematically selected, guided more by the availability of willing collaborators in regions we wished to include. Thus, the collection does not represent an epidemiological snapshot and does not represent the prevalence of those MDR pathogens in those countries.

A second key aim was to focus on isolates resistant to the polymyxins (PMX) polymyxin B (PmxB) or colistin (COL; polymyxin E). This project was funded by CARB-X as part of an antibiotic development program for novel lipopeptides active against polymyxin-resistant bacteria, and we wanted to ensure our new therapeutic was effective against contemporary clinical isolates where PMX resistance is endemic. Polymyxins are administrated as an antibiotic of last resort, particularly in LMIC’s where some even report the use of colistin as an over-the-counter (OTC) medication^36,37^; it is also used in livestock food^38^. Polymyxin resistant bacteria have generally acquired resistance against most other antibiotics by the time PmxB/COL are administered, so when polymyxin resistance develops it is usually in isolates with a high occurrence of known resistance genes. Indeed, our collection only contains two isolates resistant against PmxB/COL without any resistance against any other major classes of antibiotics, with only an additional eight isolates with limited resistance (penicillin, nitrofurantoin and fosfomycin). All other isolates resistant to PmxB/COL were also resistant to all major antibiotic classes as well, i.e. aminoglycosides, carbapenems, cephalosporins, fluoroquinolones, macrolides and tetracyclines.

While we aimed to collect mainly PmxB/COL resistant isolates, almost half (49%) of the final collection was non PMX-resistant isolates. This reflects variations in sample collection and identification across the diverse site. For collaborator sites with an existing collection of isolates (Thailand, Vietnam and Brazil) we were able to select PMX resistant isolates from an existing pool of characterised isolates. However, for collaborator sites where we initiated a new, fresh collection of isolates (Egypt, Pakistan, Nepal, Nigeria and Zambia), isolates were tested as they were collected, and we struggled to identify a sufficient number of PMX resistant isolates within the limited time frame of the project and limited number of samples collected and tested. There were also limitations in the capability of some sites to test for polymyxin resistance. We therefore included these non PMX resistant isolates within the final collection for comparison. In addition, we struggled to collect PMX resistant strains for certain species, even from pre-existing collections. Thus, the collection contains only very low numbers of PMX resistant isolates for *P. aeruginosa*, as well as limited numbers for *A. baumannii* and *E. coli* isolates.

### Processing of the isolate collection

The isolates were primarily collected at 9 sites in 8 countries, covering South America (Brazil), Northern Africa (Egypt), West Africa (Nigeria), Southern Africa, (Zambia), South Asia (Nepal and Pakistan) and Southeast Asia (Vietnam and Thailand). As part of the CARB-X project we were also able to include some isolates from the IHMA’s large isolate collection, which represents a broad geographical diversity. The focus for selection from the IHMA collection was similar to the other sites, Gram-negative bacteria with preference for PmxB/COL resistance (Table 1).

BIOCHEM: Conventional biochemical bacterial identification: Gram Stain, Triple Sugar Iron (TSI), Sulfur/Indole/Motility (SIM), Simmons Citrate and Urease Agar; E-TEST: Disk-diffusion; MS: MicroScan; BMD: Broth microdilution (mainly against colistin); RT: room-temperature; STM: Stuart semisolid transport media.

Mahidol (Thailand), NHTD (Vietnam), UNIFESP (Brazil) and IDPC (Brazil) predominantly utilized existing isolate collections which were assembled from patients in their hospital up to 5 years prior to the project start. These sites were able to select MDR and any PmxB resistant isolates using existing phenotypic information. The other sites initiated collection and characterization of the isolates over a period of 18 months, selecting relevant phenotypes for the final collection.

We received in total 677 isolates from the LMIC sites (see Table 1) and 92 LMIC isolates from the IHMA collection, which were all processed following the same workflow (see Figure S1), into a glycerol stock from single colonies. Isolates which failed to grow at the University of Queensland site were removed from the collection. All successfully growing isolates were identified (ID) by Vitek2, an automated microbiology platform widely used in hospitals around the world, and by whole genome sequencing (WGS) data. The WGS was collected by Illumina sequencing, processed into an assembled genome sequence, checked for acceptable assembly quality by *CheckM* and taxonomic classified by metagenomic analysis tool *Kraken2*.

In this process we removed any isolates which either gave undefined results in Vitek2, or conflicting results between our Vitek2 and WGS identifications, or conflicting identifications between the final site in Brisbane and the original collection site, or where the WGS identifications were inconclusive or contained a significant amount of DNA from different species (with exceptions for *Enterobacter cloacae* and *Acinetobacter baumannii*, as these often contain additional species in their corresponding complex forms).

The final list of fully validated isolates included 624 isolates from the following species: *Escherichia coli* (137), *Enterobacter cloacae complex* (29) *Acinetobacter baumannii* (82), *Acinetobacter baumannii complex* (4), *Klebsiella aerogenes* (9), *Klebsiella variicola* (3), *Klebsiella pneumoniae* and *quasipneumoniae* (260), *Pseudomonas aeruginosa* (67), *Proteus mirabilis* (5), *Serratia marcescens* (17) and *Neisseria gonorrhoeae* (11). Each of these isolates were also tested for antimicrobial susceptibility (AST) by the Vitek2 system, as well as for MIC against a panel of 44 antibiotics, determined by BMD following CLSI/EUCAST guidelines adapted to 384-well microtiter plates.

### Determining the phylogeny and sequence/sero-types

**Figure 1:**
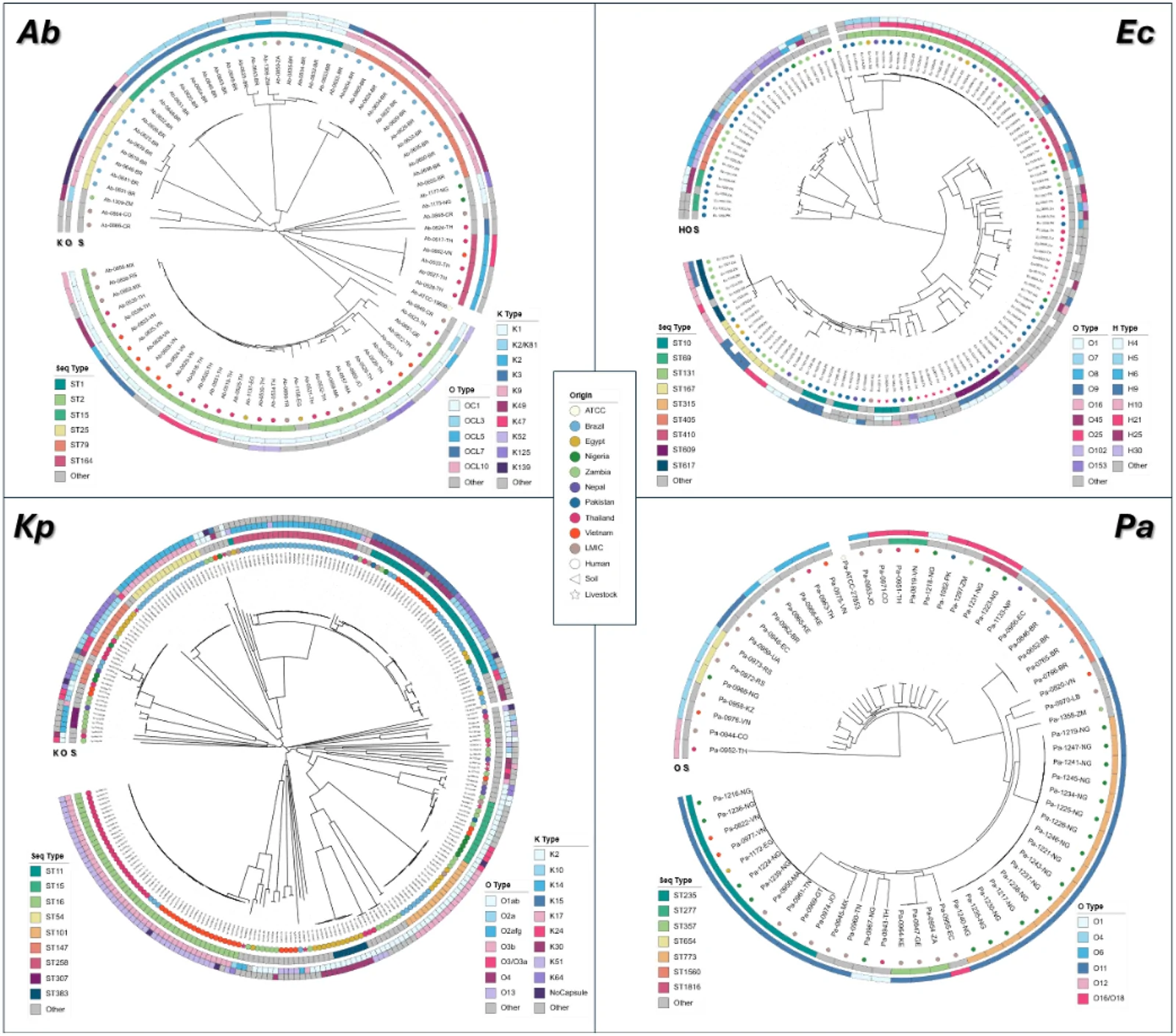
Phylogenetic analysis of high priority pathogens: *K. pneumoniae (Kp), E. coli (Ec), A. baumannii (Ab), and P. aeruginosa* (*Pa*) isolates. A maximum-likelihood phylogenetic trees were constructed based on core genome single nucleotide polymorphisms (SNPs). Reference strains from GenBank were included for comparison. The tree illustrates the genetic relationship among the isolates, highlighting distinct clades corresponding to sequence types (STs) identified through MLST.

The WGS genome sequences were analysed by Multilocus Sequence Typing (MLST) to classify each isolate to its sequence-type (ST). Similarly, sero-types were identified from the genome sequence using species specific tools, ECtyper for *E. coli*, PAst for *P. aeruginosa* and Kaptive for *K. pneumoniae* and *A. baumannii*, identifying the different liposaccharides (O) and capsule (K/H) antigen gene clusters. Figure 1 displays the phylogeny of the four main species calculated from the core sequence (using parSNP), together with the origin of the isolates, its source (human, livestock or environmental), and its sequence and sero-types.

For **E. coli** the collection contains 50 sequence types (ST), with a larger proportion of ST131, which is related to urinary and kidney infections, with serotypes (O25:H4 (22), O16:H5 (4)) similar to other collections^39^, but across different origins (Thailand, Nigeria, Egypt and Zambia).

For **K. pneumoniae** the collection contains 44 sequence types, with dominant sequence types: ST11 (35) with sero-types O2a:K64 (12) and O4:K15 (17), ST16 (48) with mainly O3b:K51 (44), ST54 with O3b:K14 (16), ST101 (17) with O1ab:K17, ST147 (16) with O2a:K64 (12) and O3/O3a:K10 (3), ST258 (26) with mainly O2afg:K107 (23), and a few ST307 (4) with O2afg:KL102. It is worth noting that the collection contains only a few capsule serotypes associated with hypervirulence (hvKp)^40^, with mainly K64 (27), K2 (7) and K5 (1) found across different regions, with higher numbers in Brazil and Egypt.

For **A. baumannii** the collection contains 18 sequence types, with dominant sequence types ST1 (8), ST2 (33), ST15 (8) ST25 (7, Brazil) and ST79 (12, Brazil).

For **P. aeruginosa** the collection contains 26 sequence types, with ST235 (12, across different regions, serotype O11) the most dominant one, with some local sequence types ST773 (16, Nigeria, serotype O11) and ST1560 (4, Brazil, serotype O4).

### Understanding the phylogeny based on multidrug resistance patterns

**Figure 2:**
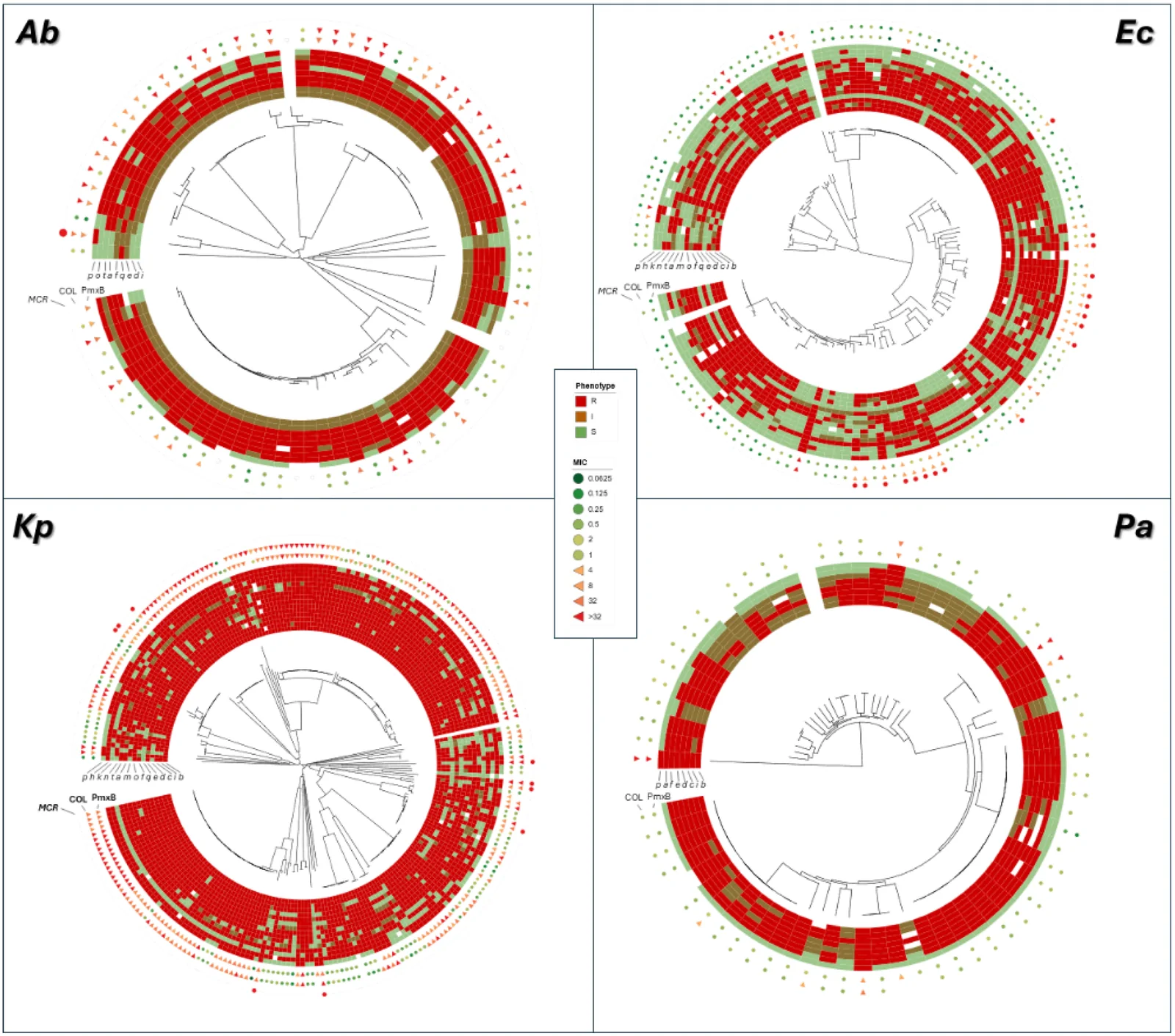
Phylogenetic analysis of high priority pathogens: *K. pneumoniae (Kp), E. coli (Ec), A. baumannii (Ab), and P. aeruginosa* (*Pa*) isolates. Ab) isolates representing the phenotypic resistance to different antibiotic classes: a) Aminoglycoside, b) Penicillin, i) Penicillin | β-lactamase Inhibitors d) Carbapenem, e) Cephalosporin, f) Fluoroquinolone, o) Folate inhibitor, m) Macrolide, c) Monobactam, n) Nitro Aromatic, k) Phenicol, h) Phosphonic Acid, p) Polymyxin, q) Quinolone and t) Tetracycline. MIC values by BMD for Polymyxin B (PmxB) and colistin (COL), as well as presence of MCR gene by AMRFinder. The maximum-likelihood phylogenetic tree was constructed based on core genome single nucleotide polymorphisms (SNPs).

The phenotypic analysis was performed with two technologies: Antimicrobial Susceptibility Testing (AST) using the Vitek2 automated system and BMD method using EUCAST compliant plate-based assays. Both technologies determine the MIC values for a range of antibiotics. The output of the Vitek2 system is however limited to the type of AST card used for analysis. Here we used the standard Gram-Negative susceptibility cards available for the Australian health sector (AST-N411 or AST-N435), with antibiotic treatment recommendations according to the Australian therapeutic guidelines. While we used the MIC values from the Vitek2 system to provide a clinical reference (see Supplementary Material), we used the ‘gold standard’ MIC values from the BMD assay as the main comparison in this study (Fig. 2).

A more detailed comparison between the AST and BMD method, indicate a strong correlation between the methods. Using the 20 antibiotic drugs, that overlap between the two methods, there is a strong correlation in the log2(MIC) values (Spearman correlation of 0.79), and a 88% agreement in the classification of R, I or S, with only 6% error rate for resistant calls. There is however a more practical difference, as the BMD method allows the testing of more, non-clinical antibiotics, especially in this project the testing of polymyxin and colistin.

Using the BMD MIC values together with the EUCAST breakpoint definition, the final collection was found to consist of predominately of multidrug-resistant (MDR) and extremely drug resistant (XDR) isolates across most of collected species, especially *A. baumannii*, *K. pneumoniae* and *P. aeruginosa* isolates (Table 2, Supplementary Figure S3).). Exceptions are *E. coli*, *E. cloacae*, and *P. mirabilis* species, which were dominated more by MDR isolates instead of XDR isolates. For *N. gonorrhoeae* only one MDR phenotype was observed.

Definition by Magiorakos et al.^41^ for MDR: resistant to ≥ 1 agent in ≥ 3 antibiotic classes; XDR: resistant to ≥ 1 agent in almost all tested antibiotic classes (susceptible in ≤ 2); PDR: resistant to every agent tested in every antibiotic class.

We tested the hypothesis as to whether polymyxin B (or colistin) resistance was indicative of multidrug-resistance against other antibiotic classes. The collected isolates indicated some dependency, as PMX resistance increased with the degree of drug-resistance, with 12% prevalence in the non-MDR isolates, 33% in the MDR set, 68% in the XDR strains, and 100% in the PDR isolates (by definition). Consistent with the hypothesis, the PMX-resistant isolates were resistant to a larger number of antibiotic classes (average of 10.9 classes) compared to PMX-susceptible isolates (average of 7.9 classes).

One other notable comment is on the comparision

### Presence of multidrug resistance and associated AMR genes

**Figure 3:**
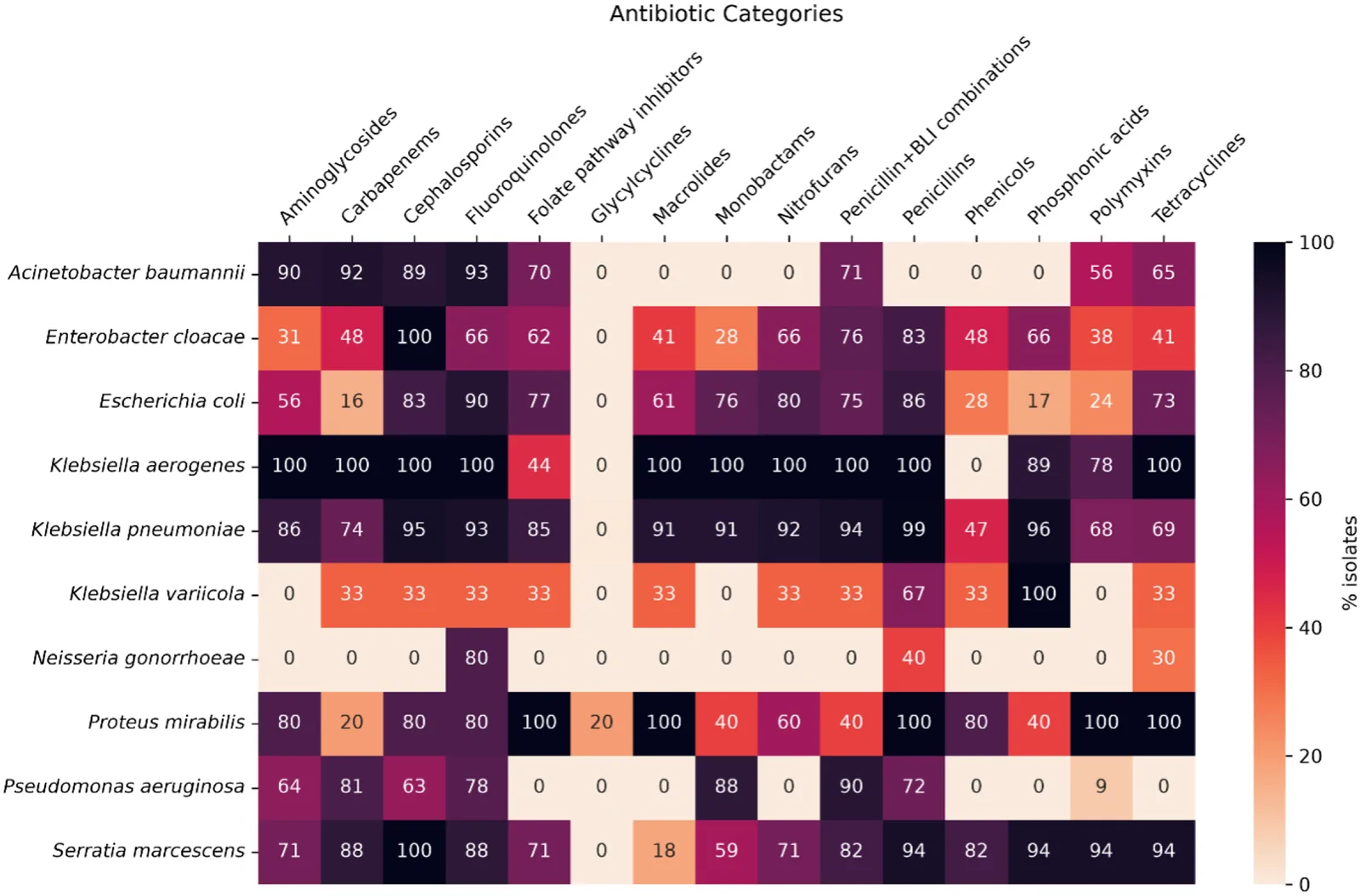
Heatmap of % resistance to specific antibiotic classes in different species, according to antibiogram profiles determined by BMD MIC testing and interpreted using EUCAST clinical breakpoints. Numbers represent the percentage of isolates for each organism, which are resistant against at least one antibiotic within an antibiotic category.

Distribution of antibiotic resistance across the bacterial strains was determined based on their antibiogram profiles as measured by BMD MIC testing and interpreted using EUCAST clinical breakpoints. Across the dataset, heterogeneous resistance patterns were observed, with several antibiogram profiles contributing disproportionately to the overall resistance burden (Fig. 3). Some resistance categories are consistently represented across multiple groups, suggesting widespread resistance to commonly used antimicrobials, whereas others are less frequent and confined to specific subsets of isolates. This variability indicates the coexistence of both prevalent and more sporadic resistance phenotypes within the population. It is worth emphasizing that the collection is not able to indicate variations in the prevalence of MDR or XDR isolates between countries, as the selection of isolates was focused on only collecting MDR isolates and polymyxin B resistance, so few susceptible, or non-MDR isolates were collected.

For *A. baumannii*, *P. aeruginosa* and *K. pneumoniae* the majority of isolates displayed resistance against some of the most common antibiotic classes, especially aminoglycosides, β-lactams and fluoroquinolones, with Klebsiella species notable in their resistance to almost all antibiotics, including macrolides, monobactams, fosfomycin and nitrofurantoin. The *E. coli* isolates, on the other hand, retained susceptibly to most antibiotics, with only dominant resistance against fluoroquinolones and β-lactams except carbapenems. The minor species in the collection, *E. cloacae*, *S. marcescens* and *P. mirabilis*, also displayed extensively resistance against most antibiotic classes, while *N. gonorrhoeae* was mainly dominated by fluoroquinolone resistance. While one of the aims was to collect PMX-resistant isolates, only 68% of *K. pneumoniae* isolates and 56% of *A. baumannii* possessed PmxB or COL resistance, with even lower levels of 24% in *E. coli* and 9% in *P. aeruginosa* The exceptions were PMX intrinsically-resistant *S. marcescens* (94%) and *P. mirabilis* (100%).

**Figure 4:**
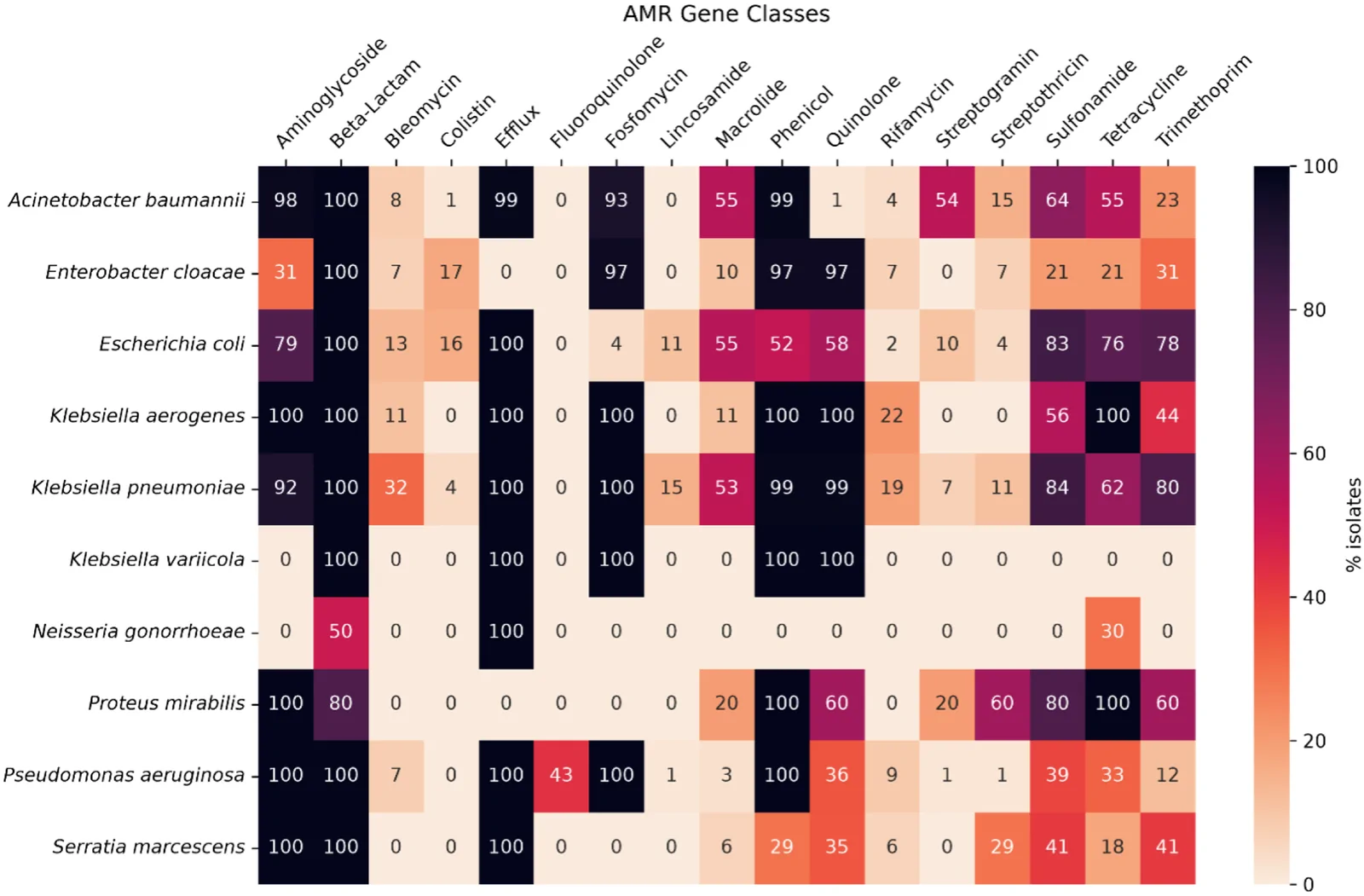
Heatmap of antimicrobial resistance (AMR) genes identified in bacterial genomes using AMR Finder. Numbers represent the percentage of isolates for each organism, with at least one identified AMR gene within each AMR Gene class.

Using the whole genome sequence data, we analysed the presence of known AMR genes using AMR Finder Plus^32^ using its latest database version. AMR Finder was able to identify the presence of AMR genes for most of the antibiotic classes in the four major species, identifying AMR genes for fosfomycin, phenicol, sulfonamides, tetracyclines and trimethoprim in most of the isolates, and AMR genes for β-lactams, aminoglycosides and efflux pump in all isolates (Fig. 4). The presence of AMR genes correlates with experimental phenotype for the majority of antibiotic classes. The striking exception is polymyxin resistance: very few isolates contained the highly publicised MCR gene for polymyxin resistance. This suggests that either the current AMR gene definition for polymyxin resistance is not complete or that polymyxin resistance is mainly induced by regulatory and protein expression, rather than mutations in the genome, chromosome or plasmids. For fluoroquinolone resistance, the AMR gene detection was more complex, involving a combination of fluoroquinolone, quinolone and efflux AMR genes.

In summary, the analysis shows a heterogeneous distribution of AMR genes across the analysed genomes, with marked differences in both the abundance and composition of resistance determinants among groups. AMR gene carriage is both quantitatively and qualitatively diverse, with frequent co-occurrence of resistance determinants within individual genomes. This pattern suggests that multidrug resistance is common within the dataset, rather than resistance being driven by single, isolated determinants. In contrast, a subset of genomes contains few or no detectable AMR genes, highlighting variability in resistance gene carriage across the population. Notably, Klebsiella showed no genes for many of the antibiotics where resistance was present in 100% of the isolates i.e. fluoroquinolones.

Comparison between phenotype and genotype, using a binary correlation (φ) between the presence of resistance and presence of AMR genes (Supplementary Figure S4), indicate a significant correlation (φ > 0.50) only for some of the antibiotic classes, such as aminoglycosides and tetracyclines, and between sulphonamides/trimethoprim’s AMR genes and DHFR inhibitor phenotypes. There some cross-correlations between fosfomycin AMR genes and carbapenem resistance, and quinolone AMR genes and a range of resistance’s including against macrolides, nitrofurans, penicillin and fosfomycin, but they might rather reflect the co-presence of these phenotypes rather than any mechanistic correlation. Notably, the correlation of colistin AMR genes (mcr) and polymyxin resistance are one of the weaker links

### Geological spread of polymyxin resistance

**Figure 5:**
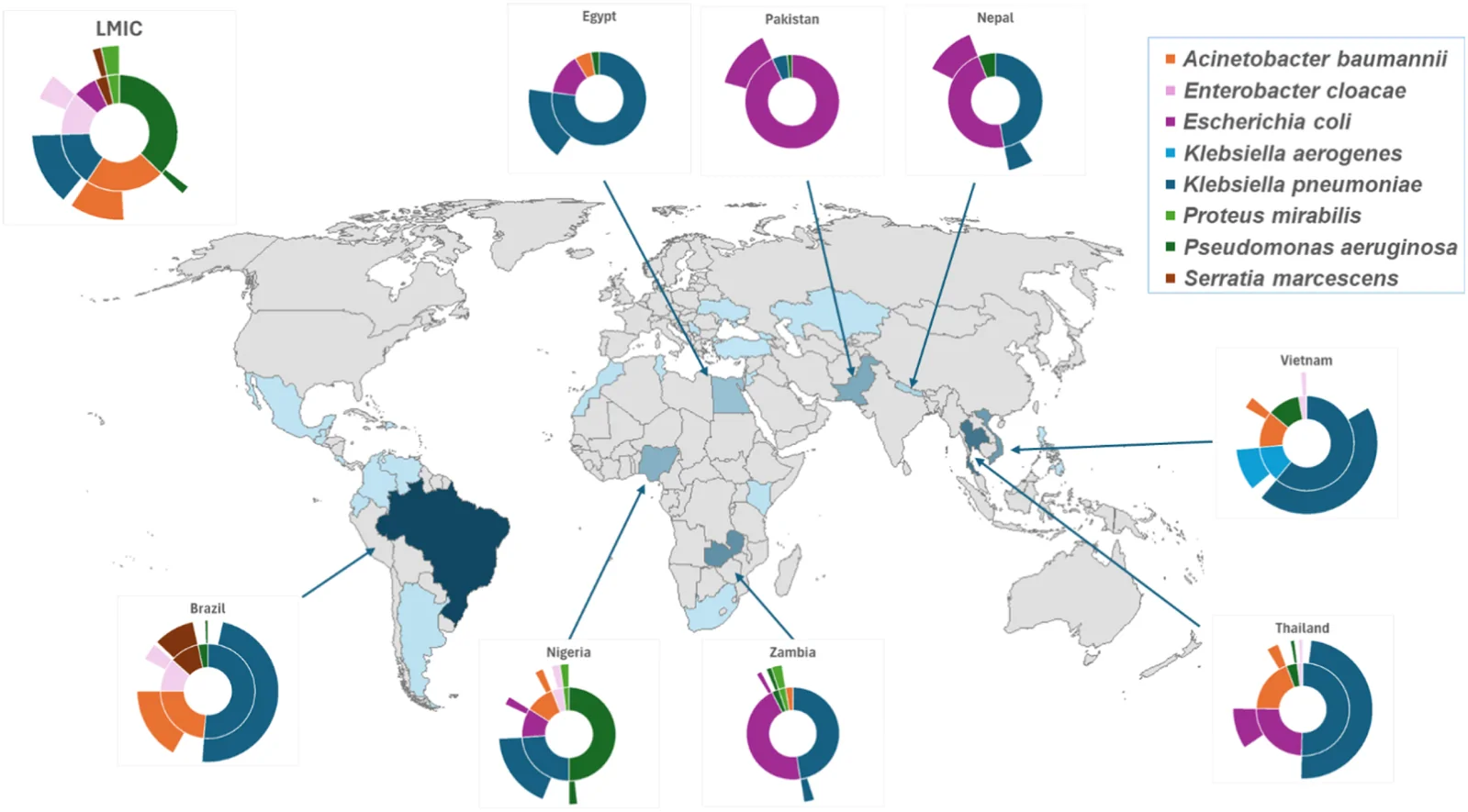
Geographic distribution of polymyxin-resistant bacterial isolates across low- and middle-income countries (LMICs). The map highlights countries contributing isolates, with sunburst charts summarising species composition. For each chart, the inner ring represents the total number of collected isolates, and the outer ring represents the number of polymyxin-resistant isolates, stratified by bacterial species. Species included are *Acinetobacter baumannii, Enterobacter cloacae, Escherichia coli, Klebsiella aerogenes, Klebsiella pneumoniae, Proteus mirabilis, Pseudomonas aeruginosa*, and *Serratia marcescens*. Country-specific charts are shown for collaborating sites (darker blue), alongside an aggregated chart representing other LMICs included in the study through the IMHA collection isolates (highlighted light blue in the map).

The range of polymyxin resistance across the different species is dependent on the country and collection site (Fig. 5), with countries like Pakistan and Nepal collecting a large proportion of *E. coli* isolates (dark purple), Nigeria a large proportion of *P. aeruginosa* (dark green), and the other sites a large proportion of *K. pneumoniae* (dark blue), with only a few *A. baumannii* isolates (orange). For Vietnam, Thailand and IHMA the collaborators were able to select from existing collections with known resistance phenotypes, so were able to successfully deliver a high rate of PMX-resistant isolates. Both sites in Brazil had a high rate of PMX-resistant *K. pneumoniae* together with some PMX-resistant *A. baumannii* isolates, and several intrinsically PMX-resistant *S. marcescens* isolates. The high rate of PMX resistant isolate collection reflects the high prevalence of PMX resistance in *K. pneumonia*^42^*e* and *A. baumannii*^43,44^ in Brazilian hospitals.

The figure illustrates substantial geographic heterogeneity in both the burden and species composition of polymyxin-resistant isolates across LMICs. While multiple countries contributed isolates, the relative proportions of bacterial species differed markedly between regions, indicating distinct local epidemiological patterns. In several countries, polymyxin resistance was presented in a limited number of contributed species, whereas the isolates provided by other countries showed a broader distribution across multiple Gram-negative pathogens. This variability potentially highlights the influence of regional antimicrobial usage practices, healthcare infrastructure, and local transmission dynamics on resistance emergence, though the inconsistent methods of isolate collection preclude any definitive conclusions.

Across most countries, *K. pneumoniae* and *A. baumannii* were consistently major contributors to polymyxin-resistant isolates, in line with their established roles as high-risk, hospital-associated pathogens. In contrast, resistance among *E. coli* and other Enterobacterales was more variable and geographically restricted, suggesting differences in selective pressure or dissemination potential. The aggregated LMIC chart further demonstrates that, when considered collectively, polymyxin resistance is distributed across a diverse range of species, underscoring the complexity of resistance patterns beyond single-country analyses.

### Comparison of polymyxin B and colistin resistance

The distribution of MIC values for COL generally matches the distributions of MIC values for PmxB, with some minor exceptions for *A. baumannii*, which displayed a higher susceptibility for PmxB compared to COL, and for *K. pneumoniae* which displayed a slight shift to lower MIC values for COL for the highly resistant isolates. In contrast, several isolates from *S. marcescens*, *P. mirabilis* and *N. gonorrhoeae* displayed significantly lower MIC values for COL compared to PmxB, with 7 of the 16 *S. marcescens* isolates susceptible to COL (MIC_COL_ <2 μg/mL) while resistant to PmxB (MIC_PmxB_ >2 μg/mL; Supplementary Figure S4). These three Gram-negative species are known to possess high level of PmxB resistance, mostly due to up-regulation of liposaccharide modifying enzymes such as L-Ara4N or pEtN. It seems that in some of the *S. marcescens* isolates this resistance mechanism is not as effective against COL, despite having very similar mode of inhibition for both polymyxins. However, the literature indicates *S. marcescens* isolates should be resistant to COL (e.g. all 43 strains tested > 32 μg/mL in one report^45^, though the prodrug colistimethate sodium was used in the assay).

**Figure 6:**
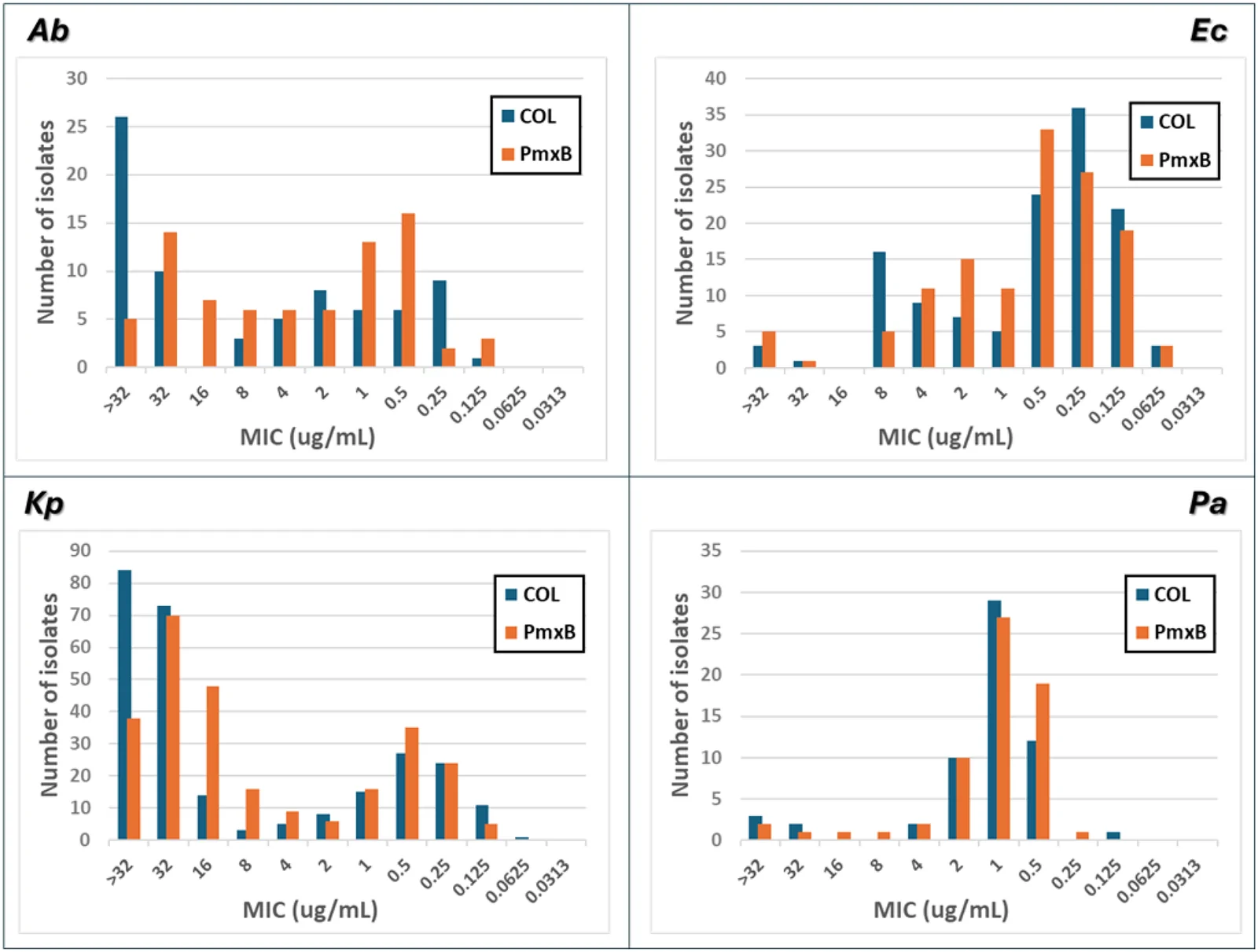
Distribution of minimum inhibitory concentrations (MICs) for colistin (COL) and polymyxin B (PmxB) among high-priority Gram-negative pathogens. Histograms show the number of clinical isolates of *Acinetobacter baumannii* (Ab), *Escherichia coli* (Ec), *Klebsiella pneumoniae* (Kp), and *Pseudomonas aeruginosa* (Pa) across MIC ranges (μg/mL) for COL (dark teal) and PmxB (orange).

We further compared the distribution of BMD-MIC distributions of COL and PmxB across four clinically important Gram-negative pathogens (Fig. 6). This revealed both species-specific susceptibility patterns and differences between the two polymyxins. *E. coli* and *P. aeruginosa* showed MIC distributions largely clustered at lower concentrations (≤ 1 μg/mL), indicating overall high susceptibility to both COL and PmxB. In contrast, *A. baumannii* displayed a broader MIC spread, including a substantial number of isolates with elevated MICs (> 8 μg/mL), suggesting reduced susceptibility or resistance in a significant subset of isolates. *K. pneumoniae* demonstrated the most concerning profile, with a pronounced shift towards higher MIC values for both polymyxins. A large proportion of *K. pneumoniae* isolates fall in the ≥ 16–32 μg/mL range, consistent with widespread polymyxin resistance in this species. However, the analysis reveals a similar bimodal distribution across all species, with the main distribution of MIC values for most species between 0.25 and 8 μg/mL (covering the clinical breakpoint at 2 μg/mL), and a second distribution of highly resistant isolates between 16 and > 32 μg/mL.

Notably, while both PmxB and COL follow similar overall trends within each species, subtle differences in MIC distributions are evident, with PmxB tending to show slightly lower MICs than COL in some organisms, particularly *E. coli* and *P. aeruginosa*.

The MIC data highlights the heterogeneity of polymyxin susceptibility among high-priority pathogens and underscore the ongoing challenge of treating infections caused by multidrug-resistant *A. baumannii* and *K. pneumoniae*. The overlapping but non-identical MIC profiles of colistin and polymyxin B suggest that, although closely related, their in vitro activities are not always interchangeable, reinforcing the importance of species-specific and drug-specific susceptibility testing in clinical and surveillance settings.

## DISCUSSION

This study represents one of the most comprehensive genomic investigations to date of polymyxin-resistant Gram-negative pathogens circulating across LMICs. The analysis of 624 clinical isolates from 28 LMICs using whole-genome sequencing, phylogenetic reconstruction, and phenotypic susceptibility testing addresses a critical evidence gap in regions that bear a disproportionate burden of antimicrobial resistance (AMR) yet remain underrepresented in global surveillance initiatives. Our findings provide new insight into the diversity, dissemination, and genetic architecture of polymyxin resistance in settings where therapeutic options are already severely constrained.

The predominance of polymyxin resistance in LMICs has profound clinical and public health implications. In many of these settings, polymyxins represent one of the final therapeutic options for treating infections caused by carbapenem-resistant organisms due to its availability and affordability. The spread of polymyxin-resistant, high-risk clones therefore significantly narrows already limited treatment choices and increases the risk of untreatable infections. Furthermore, constrained access to advanced diagnostics and limited antimicrobial stewardship infrastructure in LMICs may facilitate unrecognised transmission and inappropriate antimicrobial use, further selecting for resistant strains.

The identification of 12 distinct bacterial species among polymyxin-resistant isolates highlights the broad and concerning taxonomic distribution of resistance to last-line antibiotics. Genetic analyses focusing on *K. pneumoniae, E. coli, A. baumannii*, and *P. aeruginosa* revealed that resistance is dominated by lineages previously implicated in global outbreaks and healthcare-associated infections^46–48^. The presence of closely related sequence types across geographically distant LMICs strongly suggests international dissemination of epidemic clones rather than independent, de novo emergence of resistance. This pattern mirrors global trends observed for carbapenem-resistant organisms and underscores the capacity of successful bacterial lineages to exploit weaknesses in infection prevention and control systems, particularly in resource-limited healthcare settings^49,50^.

Phylogenetic analyses provide further evidence that horizontal gene transfer plays a substantial role in shaping resistance landscapes in LMICs. The distribution of resistance determinants across unrelated sequence types suggests repeated acquisition events rather than vertical inheritance alone. Such genetic fluidity increases the likelihood that polymyxin resistance will continue to emerge in new genetic backgrounds, complicating surveillance and limiting the effectiveness of clone-targeted interventions^51,52^. In contrast, the clustering observed in *A. baumannii* and *P. aeruginosa* reflects the success of highly adapted hospital-associated clones, which are known to persist in healthcare environments and rapidly accumulate resistance-conferring mutations^53^.

The genomic architecture of polymyxin resistance observed in this study reflects the complexity and adaptability of Gram-negative pathogens. From the whole genome data analysis, only 40 (14%) of the 279 polymyxin resistant isolates in our collection (excluding any intrinsically resistant species) could be predicted based on mobilized colistin resistance (MCR) genes, with 25 of those isolates from livestock samples and 15 from human samples. While still low, the current prevalence of mobile genetic elements in *Enterobacterales* is concerning, as they potentially facilitate rapid interspecies transmission and long-range dissemination across clinical and environmental reservoirs [15,27]. For the remaining, non-MCR polymyxin resistant isolates, they are most likely related to changes in regulatory pathways, such as the *pmrAB* and *phoPQ* two-component systems, which are not detectable by the standard DNA sequencing technologies applied in this study. The coexistence of these mechanisms within and across lineages indicates that polymyxin resistance is frequently the result of layered evolutionary processes, driven by intense antimicrobial selective pressure^54,55^.

Phenotypic antimicrobial susceptibility testing using both VITEK 2 and BMD assays largely corroborated bioinformatic predictions, supporting the reliability of whole-genome sequencing–based resistance profiling. However, instances of discordance between genotypic and phenotypic results highlight ongoing challenges in accurately predicting resistance phenotypes. These findings emphasise the need for improved predictive frameworks, alternative sequencing strategies, and continued harmonisation of laboratory methods, alongside cautious interpretation of automated susceptibility results—particularly in resource-limited settings.

From a public health perspective, our findings underscore the urgent need to strengthen genomic surveillance capacity in LMICs. WGS-based approaches enable early detection of emerging resistance mechanisms, tracking of epidemic clones, and informed decision-making for infection prevention and control. While cost, infrastructure, and technical expertise remain barriers, recent advances in sequencing technologies and international collaborative networks offer feasible pathways for expanding genomic capacity in resource-limited settings.

Several limitations should be acknowledged. Although this study includes isolates from a wide range of LMICs, sampling was selective and does not capture national epidemiological distributions or community-level transmission dynamics. Additionally, incomplete clinical metadata limited our ability to directly link genomic findings to patient outcomes, antimicrobial exposure, or treatment failure. Future longitudinal studies incorporating systematic sampling, richer epidemiological data, and environmental reservoirs will be essential to fully elucidate transmission pathways and inform targeted interventions^23,56^.

In conclusion, this large-scale genomic analysis demonstrates that polymyxin resistance in LMICs is driven by a combination of clonal expansion of high-risk lineages and horizontal gene transfer across key Gram-negative pathogens. Understanding these evolutionary trajectories is essential for anticipating future resistance threats and for informing strategies aimed at preserving the effectiveness of last-line antibiotics. This data supports the continued use of integrated genomic and phenotypic approaches for resistance surveillance and highlights the need for standardised susceptibility testing and resistance interpretation across laboratories, with polymyxin resistance particularly challenging but critical to accurately assess.

## Supplementary Material

Supplementary Files

This is a list of supplementary files associated with this preprint. Click to download.

- LMICCollectionSupplementary.pdf

## Figures and Tables

**Table 1:** Summary of isolate samples with identification and characterisation at the collaborator site and details of shipment.

Collaborator Site		Years	Total Isolates	Shipment	ID	AST
Mahidol	Faculty of Medicine Siriraj Hospital, Mahidol University, Bangkok, Thailand	2017–2022	100	STM swabs (RT)	BIOCHEM	E-TEST BMD
NHTD	Department of Microbiology, National Hospital for Tropical Diseases, Hanoi, Vietnam	2019–2022	65	Lyophilised (RT)	MALDI-TOF	VITEK E-TEST BMD
IDPC	Instituto Dante Pazzanese de Cardiologia, São Paulo, Brazil	2020–2021	15	Charcoal swabs (RT)	MALDI-TOF	VITEK BMD
UNIFESP	Institute of Biomedical Sciences, Federal University of São Paulo - UNIFESP, São Paulo, Brazil	2017–2021	149	Charcoal swabs (RT)	MALDI-TOF	
NIBGE	National Institute for Biotechnology and Genetic Engineering, Faisalabad, Pakistan	2022	80	Agar slants (RT)		E-TEST
NMH	Department of Laboratory Medicine and Pathology, Nepal Mediciti Hospital, Latipur, Nepal	2022	54	Agar slants (RT)	BIOCHEM	E-TEST
SRTACity	City of Scientific Research and Technology Applications, Alexandria, Egypt	2022	38	Agar slants (RT)	VITEK	VITEK E-TEST
ICL	Innovation Curae Limited, Lagos, Nigeria	2022	76	Agar slants (RT)	API 20E/20NE	MS BMD
CIDRZ	Centre for Infectious Disease Research in Zambia, Lusaka, Zambia	2022	100	Charcoal swabs (RT)	VITEK	VITEK
IHMA	IHMA Europe Sàrl, Route de l'Ile-au-bois 1A 1870 Monthey/VS, Switzerland	2014 2019	92	Glycerol stock (dry ice)		BMD

**Table 2:** Number of multidrug-resistant (MDR), extremely drug resistant (XDR) and pandrug-resistant (PDR) for each organism in the collection.

Organism	non-MDR	MDR	XDR	PDR
*A. baumannii*	5	28	52	
*E. cloacae*		22	7	
*E. coli*	8	115	13	
*K. aerogenes*		1	8	
*K. pneumoniae*	2	72	177	8
*K. variicola*	1	2		
*N. gonorrhoeae*	9	1		
*P mirabilis*		4	1	
*P aeruginosa*	11	11	43	2
*S. marcescens*		8	9	

## Data Availability

The whole-genome project was deposited at the NCBI GenBank under BioProject number **PRJNA1322038**, together with the VITEK 2 AST determination.

## Code availability:

The data analysis, genomic processing codes is available upon request to the authors.
